# Label-Free Electrochemical Detection of the Specific Oligonucleotide Sequence of Dengue Virus Type 1 on Pencil Graphite Electrodes

**DOI:** 10.3390/s110605616

**Published:** 2011-05-26

**Authors:** Elaine Souza, Gustavo Nascimento, Nataly Santana, Danielly Ferreira, Manoel Lima, Edna Natividade, Danyelly Martins, José Lima-Filho

**Affiliations:** 1 Laboratory of Immunopathology Keizo Asami (LIKA), Universidade Federal de Pernambuco-UFPE, Av. Professor Moraes s/n, 50670-901 Recife, PE, Brazil; E-Mails: galvesn23@gmail.com (G.N.); nataly_amorim@yahoo.com.br (N.S.); daniellylsantos@hotmail.com (D.F.); 2 Computer Science Institute, Universidade Federal de Pernambuco-UFPE, Av. Professor Moraes s/n, 50670-901 Recife, PE, Brazil; E-Mails: manoeleusebio@gmail.com (M.L.); edna.barros@gmail.com (E.N.); 3 Department of Biochemistry, Universidade Federal de Pernambuco-UFPE, Av. Professor Moraes Rego s/n, 50670-901 Recife, PE, Brazil; E-Mails: bruneska@prospecmol.org (D.M.); joseluiz60@mac.com (J.L.-F.)

**Keywords:** dengue virus, nucleic acid biosensor, guanine oxidation

## Abstract

A biosensor that relies on the adsorption immobilization of the 18-mer single-stranded nucleic acid related to dengue virus gene 1 on activated pencil graphite was developed. Hybridization between the probe and its complementary oligonucleotides (the target) was investigated by monitoring guanine oxidation by differential pulse voltammetry (DPV). The pencil graphite electrode was made of ordinary pencil lead (type 4B). The polished surface of the working electrode was activated by applying a potential of 1.8 V for 5 min. Afterward, the dengue oligonucleotides probe was immobilized on the activated electrode by applying 0.5 V to the electrode in 0.5 M acetate buffer (pH 5.0) for 5 min. The hybridization process was carried out by incubating at the annealing temperature of the oligonucleotides. A time of five minutes and concentration of 1 μM were found to be the optimal conditions for probe immobilization. The electrochemical detection of annealing between the DNA probe (TS-1P) immobilized on the modified electrode, and the target (TS-1T) was achieved. The target could be quantified in a range from 1 to 40 nM with good linearity and a detection limit of 0.92 nM. The specificity of the electrochemical biosensor was tested using non-complementary sequences of dengue virus 2 and 3.

## 1. Introduction

Dengue is a disease caused by four serologically related viruses called dengue virus (DENV) type-1, -2, -3 and -4. They are members of *Flaviviridae* family and have a single-stranded positive-sense RNA genome of approximately 11 kb. Infection with DENV may cause an acute “influenza-like” febrile disease called classic dengue fever (DF), or progress at the time to dengue hemorrhagic fever (DHF) characterized by bleeding and plasma leakage [1] and dengue shock syndrome (DSS) [2].

The mosquito *Aedes aegypti* (Diptera: Culicidae) is responsible for the spread of diseases such as yellow fever and dengue among humans because of its role as a vector [3]. Yellow fever and dengue are endemic to Central and South America, Asia and Africa [4], and they are important public health problems in the tropic and subtropic areas, which encompass many resource-poor countries. Brazil currently accounts for the majority (80%) of dengue cases reported in Latin America, with co-circulation of three serotypes (DENV 1–3) in most of the country and sporadic epidemic waves in several urban areas [5–7].

Dengue virus is difficult to control due to massive urbanization, overpopulation, continually increasing travel, and failure to maintain effective control programs against the mosquito vector [8]. To date the only option for controlling dengue virus transmission in the human population has been reduction of the population density of the mosquito vectors of dengue [9]. Diagnosis for the detection of dengue infection has been carried out by IgM capture ELISA, virus isolation in mosquito cell lines and live mosquitoes, dengue specific monoclonal antibodies, PCR (polymerase chain reaction) and reverse-transcription polymerase chain reaction (RT-PCR) assays [10], which usually are reportable after 5 or 7 days. Because of the importance of early diagnosis, development of a procedure for detection of viral nucleic acids in a biological sample is highly desirable. The use of a biosensor for the detection of nucleic acid sequences based on nucleic acid hybridization processes can greatly reduce the assay time and simplify the protocol, allowing detection of nucleic acid sequences almost in real time [11–14].

Due to their high sensitivity, small dimensions, low cost, and compatibility with microfabrication technology electrochemical transducers are often used for detecting hybridization events [12,15,16]. Sensitive electrochemical signaling strategies are based on the direct or catalyzed oxidation of nucleic acid nucleotides, as well as the redox reactions of reporter molecules in the indirect detection using chemical indicators [17,18]. The most common indicators for this purpose are heterocyclic dyes (e.g., ethidium bromide and methylene blue) and organometallic complexes (mainly Co, Fe, Os, Pt and Ru) [16].

A technique using direct guanine oxidation signal on carbon paste electrodes in which PCR amplicons of specific genotypes of the factor V Leiden mutation were identified has been previously reported [19]. In this paper, we describe a simple, inexpensive and stable electrode for the possible detection of the dengue virus type 1. It is a nucleic acid-based electrochemical biosensor using the partial sequence of dengue virus type 1 as a probe that allows differentiation of complementary from non-complementary sequences, and utilizing the annealing temperature for the hybridization process.

## 2. Experimental Section

### 2.1. Materials and Reagents

Tris base was obtained from Promega (USA), and sodium acetate was obtained from Sigma (USA). UltraPure distilled water was purchased from Invitrogen (USA), and reagents were of analytical grade. All DNA oligonucleotides (probe, target and non-complementary sequences) were synthesized by Integrated DNA Technologies (Brazil). *Probe*: Dengue virus type specific 1 (TS-1P)_ 5′CGTCTCAGTGATCCGGGG 3′; *Target*: Dengue virus type specific 1 (TS-1T)_ 5′ CCCCGGATC ACTGAGACG 3′; *Non-complementary*: Dengue virus type specific 2 (TS-2 NC)_ 5′ CTGTTCATGG CCCTTGTGG CG 3′; Dengue virus type specific 3 (TS-3 NC)_ 5′ GCTCTGTCTCATGATGATGTT A 3′; PolyG-NC_ 5′ GGGGGGGGGGGGGGGGGGGG 3′. The oligonucleotide stock solutions and dilute solutions were prepared with 0.5 M acetate buffer (pH 4.8) and kept frozen.

### 2.2. Bioinformatics

The complete genomes of dengue virus types 1, 2 and 3, corresponding to GenBank_ accession numbers U88536.1, U87411.1 and AY099336.1, respectively, were obtained from the National Center for Biotechnology Information (NCBI) database. The probe specific for dengue type 1 (TS-1P) was constructed based on Duebel [20], and the sequence was aligned using the CLC Combined Workbench v 3.6.1 software. Target sequence (TS-1T) and non-complementary (TS-2 NC and TS-3 NC) sequences were also constructed using this software.

### 2.3. Apparatus

Electrochemical analysis was carried out with the Autolab PGSTAT apparatus (Metrohm Autolab, The Netherlands). Voltammetric signals were measured using a system consisting of two electrodes [21] Pencil graphite (type 4B) was used as the working electrode, and the reference electrode was screen-printed using Ag/AgCl ink (Electrodag-Acheson, USA) under gold wire and then dried at 60 °C. The experiments were performed in triplicate.

### 2.4. Preparation of the Graphite Electrode

Pencil lead (type 4B, total length of 3 cm and diameter of 2.5 mm) typically composed of natural graphite was used as the pencil graphite electrode (PGE). The graphite was polished with an emery-impregnated disc to obtain a smooth surface. The body of the pencil was coated well with silicone, resulting in a free area for immobilization of 28.50 mm^2^. Afterwards, the graphite electrode was washed with ultra-pure water to remove possible contaminants on the surface of the electrode. The polished surface of the working electrode was then activated by applying a potential of 1.8 V for 5 min [22]. The working electrode was fixed vertically and immersed in a solution of 0.5 M acetate buffer (pH 4.8) containing the TS-1P probe.

### 2.5. Immobilization of the DNA Probe

The TS-1P probe was immobilized on the activated electrode by applying 0.5 V [20] to the electrode for 1–10 min in 0.5 M acetate buffer solution (pH 4.8) containing oligonucleotides at different concentrations. The electrode was then rinsed with Tris-HCl (pH 7.0).

### 2.6. Hybridization of Target Sequence

The working electrode with the immobilized probe was immersed in a solution containing the target oligonucleotides in acetate buffer, pH 4.8, and incubated at 57 °C for 3 min. The electrode was then washed with Tris-HCl (pH 7.0) to remove non-hybridized sequences. The same protocol was applied for the interaction of the probe with non-complementary sequences.

### 2.7. Electrochemical Analysis

To obtain electrochemical signal data, the biosensor surface with hybridized probe and target nucleic acid was immersed in an electrolytic cell containing Tris-HCl (pH 7.0), and attached to the Autolab potentiostat, equipped with GPES 4.9 software. Differential pulse voltammetry was applied to detect electrochemical signals, whereby a potential sweep was applied between 0.5 and 1.2 V, at a pulse amplitude of 50 mV and scan rate of 20 mV/s. The raw data were treated using the GPES software with a moving average baseline correction using a “peak width” of 0.01.

### 2.8. Statistical Analysis

Experimental data were analyzed using the Statistica 8 software by non-parametric tests. To evaluate statistical significance, the Mann–Whitney U test was used for the comparison of two unpaired groups. The Kruskal-Wallis test was used for the comparison of multi independent group data. A level of p < 0.05 was considered significant.

## 3. Results and Discussion

### 3.1. Preliminary Investigation

Research has demonstrated the utility of graphite electrodes [23] in the construction of electrochemical biosensor utilizing oligonucleotides [12,15]. The good sensitivity of the carbon electrodes in detecting the oxidation of nucleic acids makes them widely useful in DNA research, especially when inexpensive pencil graphite electrode is used [24]. The immobilization of nucleic acids on the surface of the electrode occurs by adsorption, which is considered the simplest method, and it does not require any special reagents or nucleic acid modifications. The smoothed surface of the pencil graphite was pretreated applying a potential (*vs.* Ag/AgCl reference electrode) of 1.8 V for 5 min. The pretreatment of the carbon surface increases its roughness and hydrophilicity, facilitating the adsorption of the probe on the electrodes [23].

The covalent immobilization of the probe is more stable, but the electrode surface may contain groups capable of reacting with and fragmenting the oligonucleotide molecule, damaging and changing its original structure [25]. The immobilization and hybridization are based on the electrochemical oxidation of guanine and adenine groups, which occurs with different electron transfer reaction rates [26,27]. We detect the oxidation of guanine that it is the most redox active nitrogenous base in DNA strands [12], which has been shown in some works as peaks of +0.93–1.0 V [26,28].

**Figure 1.**
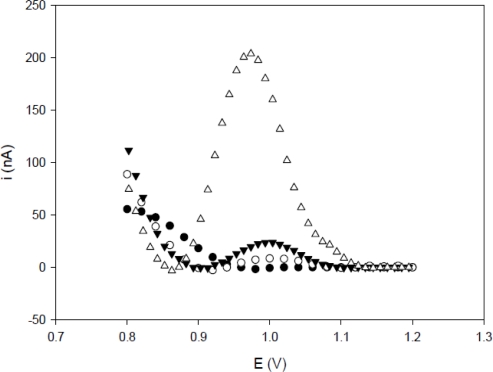
The differential pulse voltammograms of guanine oxidation on (•) non-activated PGE, (○) activated PGE, (▾) TSF-1P (1 μM) immobilized on non-activated PGE and (Δ) TSF-1P (1 μM) immobilized on activated PGE. Voltammetric conditions: scanning potential steps, 20 mV/s; potential amplitude, 50 mV.

The influence of the pretreatment of the PGE at 1.8 V was investigated. Figure 1 shows no significant difference between the current peaks of activated PGE and TS-1P (1 μM) non-activated PGE. This observation indicates that the adsorption of nucleic acid did not occur. While the signal of the TS-1P (1 μM) on activated PGE were significantly higher than the signals of three other electrodes, due to the immobilization of the oligonucleotides containing guanines. These results demonstrated that applying a potential (1.8 V) on electrodes allows the adsorption of oligonucleotides.

### 3.2. Detection of Guanine Oxidation via the Differential Pulse Voltammetry

The immobilization of the probe on the activated PGE involved the application of a potential of 0.5 V for 5 min [12,23,29,30]. This potential enhances the stability of the immobilized probe through the electrostatic attraction between the positively charged carbon surface and the negatively charged hydrophilic sugar-phosphate backbone with the bases oriented toward the solution ready to hybridize with the target [27].

**Figure 2.**
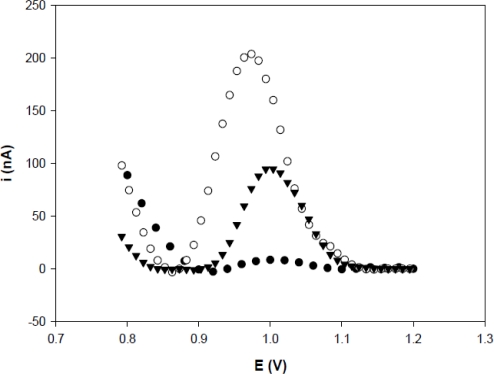
The differential pulse voltammograms of guanine oxidation on (•) an activated PGE, (▾) TS-1T (1 μM) immobilized on an activated PGE and (○) TS-1P (1 μM) immobilized on an activated PGE. Voltammetric conditions: Scanning potential steps, 20 mV/s. Potential amplitude, 50 mV.

The influence of the number of guanine in the probe was investigated immobilizing 1 μM of TS-1P (with seven guanines) and TS-1T (with five guanines) on different working electrodes. Figure 2 shows that the TS-1P probe (206 ± 4.3 nA) has a current peak two times larger than that of TS-1T (84.7 ± 5.0 nA). These results were similar those obtained by Pournaghi-Azar *et al*. [31] with a biosensor for interleukin-2. They also observed that current peak was higher for the sequence with seven guanines compared to that with one guanine.

The TS-1T sequence was chosen as a target sequence, because it is present in the dengue virus genome. TS-1P is the sequence complementary to the RNA present in the virus, and thus used as the probe for construction of the biosensor. The data obtained were analyzed using the CLC Combined Workbench v 3.6.1 and NCBI Software.

### 3.3. Effect of Immobilization Time on the Probe

**Figure 3.**
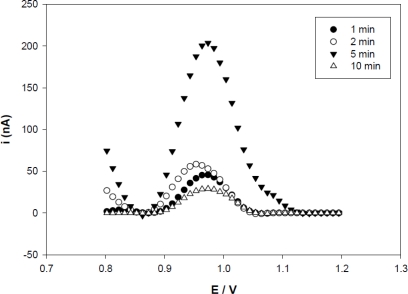
The differential pulse voltammograms of guanine oxidation at TSF-1P (1 μM) immobilized activated for different times (•) 1 min, (○) 2 min, (▾) 5 min and (Δ) 10 min. Voltammetric conditions: scanning potential steps, 20 mV/s; potential amplitude, 50 mV.

Probe immobilization is a crucial step for fabricating an electrochemical biosensor [18]. In Figure 3, the results obtained with anodic differential pulse (ADP) show the guanine oxidation signal increasing with time up to 5 min and decreasing significantly after that. This decrease can be attributed to the massive accumulation of the probe [29] on the graphite electrode, leading to an overlapping of the probes and lower availability of guanine bases. These results showed that probe immobilization could be achieved within 1 to 10 min. However, five minutes was suggested as the optimal time for probe immobilization since the electrode achieved a higher peak current. This result also was found in the systems for gene detection of interleukin-2 [29], hepatitis B [15] and electrochemical detection for human papilloma virus [23].

### 3.4. Effect of the Probe Concentration

**Figure 4.**
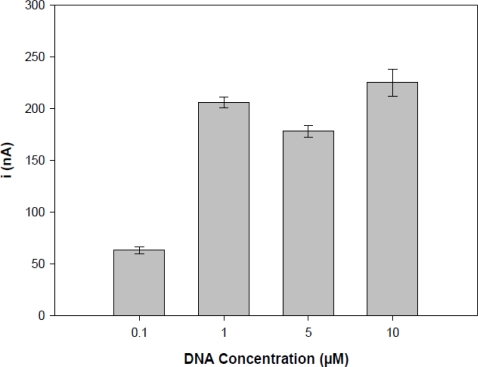
Current peaks of the guanine oxidation signal with different concentrations of the TS-1P modified activated PGE (0.1 μM, 1 μM, 5 μM and 10 μM). The results were plotted using the means of experiments performed in triplicate.

The effect of probe concentration is shown in Figure 4, it was carried out by differential pulse voltammogram after 5 min of oligonucleotides (TS-1P) immobilization using a potential of 0.5 V in acetate buffer (pH 4.8) containing different concentrations of probe (0.1 to 10 μM).

The results showed that oxidation peak currents increased from 0.1 μM up to 1.0 μM with TS-1P. However, since the 1 μM the signal is stabilized, it was the chosen concentration to carry on further experiments. The results, analyzed with STATISTICA 8.0 using non-parametric tests (Kruskal-Wallis), showed that 1 μM–10 μM results were statistically identical. Thus, the concentration of the 1 μM and 5 min were considered the best experimental conditions for electrode preparation. Next, the study sought to build a working electrode, with an area completely filled with the oligonucleotide sequence (probe).

### 3.5. Hybridization Detection

The electrodes modified with nucleic acid (polynucleotide) identify the sequence of complementary bases, through the formation of a double helix. This identification is effective and specific showed in the presence of other non-complementary sequences. When the target oligonucleotide sequence corresponds to the probe (based on the complementarily principle stating that G pairs with C and A with T or U), a hybrid is formed (probe-target).

The development of biosensor DNA or RNA consists of three steps: (a) adsorption of the oligonucleotide probe; (b) hybridization between the probe and its complementary sequence (the target) to form the hybrid; and (c) transduction [27]. The hybridization devices for biological detection of specific oligonucleotides sequences can be fabricated by applying a constant potential [32], incubating at a specific annealing temperature and at room temperature [12,33].

There have been various reports on label-free oligonucleotide biosensors for hybridization detection [12,22]. The oligonucleotide-modified electrode is dipped into a solution of target (DNA or RNA) to test its nucleotide sequence. When hybridization occurs, there is a decrease in electrochemical signals due to the interaction of free guanines of the probe with complementary cytosine bases present in the target sequence [22,27], the principle was used in this work. This is because of less guanine bases are availabile in the hybrid form for oxidation. Another strategy for the detection of guanine oxidation is the use of immobilized inosine-substituted (for guanine) or guanine-free probes [22], where there is a direct detection of oligonucleotide hybridization by the appearance of the oxidation signal due to the presence of guanines in the target sequence [27].

The hybridization experiments were performed in a microtube (0.2 mL), containing the probe-modified PGE, which were immersed in acetate buffer containing a known amount of complementary target oligonucleotides at a specific annealing temperature. Detection of target oligonucleotides was monitored with the formation of a guanine current peak on the probe immobilized on PGE with or without the target sequence.

**Figure 5.**
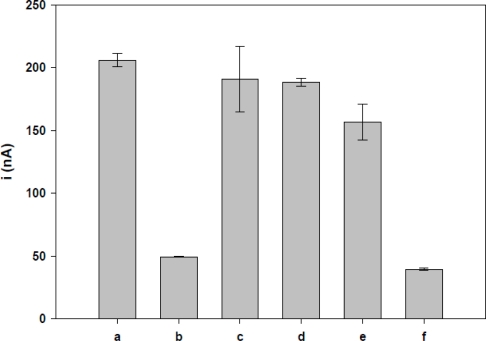
The differential pulse voltammograms of guanine oxidation at **(a)** TS-1P (1 μM) immobilized on activated PGE before hybridization, **(b)** TS-1P (1 μM) immobilized on activated PGE after hybridization with complementary TS-1T (40 nM), **(c)** with PolyG-NC (40 nM), **(d)** with TS-2 NC (40 nM) **(e)** with TS-3 NC (40 nM) and **(f)** mixture of complementary and non-complementary (PolyG-NC, TS-2 NC and TS-3 NC) (40 nM each). Voltammetric conditions: Scanning potential steps, 20m V/s. Potential amplitude, 50 mV.

The results are shown in the voltammogram for guanine oxidation (0.97 V in 20 mM Tris-HCl buffer solution, pH 7) on the probe-modified and activated PGE, before and after hybridization in solutions of complementary and non-complementary oligonucleotides (Figure 5). The current peak generated by guanine oxidation was monitored by the detection of the target oligonucleotides. The probe contains seven guanine bases, and after hybridization with the target sequence it contains only five guanines, thus there is a significant reduction in the guanine oxidation peak.

The current peak of the hybridization at the concentrations 0.5, 1, 10, 20, 30, 40 and 100 nM was statistically different from that observed with the TS-1P sequence at the concentration of 1.0 μM. The results showed that the complementary sequence could produce hybridization, causing a decrease in the guanine oxidation signal.

### 3.6. Selectivity Study

The highest guanine oxidation signal was observed with TS-1P immobilized on PGE, which has seven guanine bases present in the oligonucleotides. Experiments with non-complementary oligonucleotides including TS-2 NC, TS-3 NC and PolyG-NC (Figure 5) were performed to evaluate the selectivity of electrochemical nucleic acid biosensor. The interaction between these non-complementary oligonucleotides and probe did not lead to a significant decrease in the guanine oxidation due to absence of entire hybridization. So, the current peak of hybridized with a non-complementary sequence showed values similar to that observed following TS-1P. The selectivity demonstrates that the sequence (TS-1T) could form a double helix with the complementary oligonucleotides, causing a significant decrease in the guanine oxidation current peak.

These results confirmed that the electrochemical detection in the present study successfully distinguished complementary sequences from non-complementary sequences, using the annealing temperature to perform the hybridization. The selectivity of the biosensor was also examined in a sample containing both complementary and non-complementary sequences in similar proportions. The mixture showed a peak current similar to immobilized TS-1P after hybridization with complementary sequence. This demonstrates that the presence of non-complementary samples did not interfere in the specificity of the biosensor. These results are important for the construction of the device, because the system can be better controlled by regulating temperature than by other means such as the application of a potential.

### 3.7. Detection Limit

**Figure 6.**
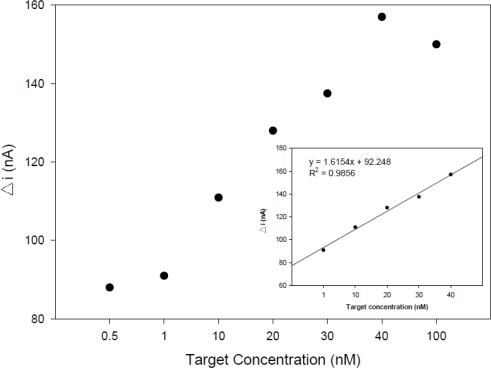
Plot of ΔI (difference of guanine oxidation signal of the probe-modified PGE in the absence and presence of the target) *vs.* target concentration. Inset: related calibration graph at concentration range 1–40 nM for complementary target.

The detection limit was determined using the synthetic target sequence. The difference in the guanine oxidation signal of the probe-modified PGE in the absence and presence of complementary sequence (ΔI) increased when the target concentration increased and leveled off at a concentration of 40 nM. As seen in the inset of Figure 6, the signal was linear between 1 and 40 nM with a correlation coefficient of 0.9856 for complementary target.

The detection limit and quantitation limit values were calculated using the following equations: y_LOD_ = 3s/m and y_LOQ_ = 10 s/m, respectively [34], where s is signal of the standard deviation of the blank (0.5 nM), and m is the slope (1.6154) of the related calibration line. The regression equation was ΔI = 1.6154C + 92.248 (C, nM; ΔI, A), thus the detection limit (0.92 nM) and quantitation limit (3.09 nM) were measured, and both values confirmed the sensitivity of the device. The within day and between day reproducibility (R.S.D.%) results of the signal (three independently probes) measured at 40 nM of target were 0.61% and 1.72%, respectively, indicating a remarkable reproducibility of the detection method.

Compared with other nucleic acid biosensors obtained under the same immobilization conditions (adsorption), ours displayed good sensitivity with a hybridization time of 3 minutes. In Table 1, the biosensor proposed in the present work has a low detection limit compared with the others. High-sensitivity nucleic acid detection is essential for clinical diagnosis, pathology, and genetics [35]. The use of an electroactive indicator can provide a slightly lower detection limit when compared with guanine oxidation. However, label-free monitoring based on guanine moiety oxidation signal of target in biosensors are easily manipulated, have fast responses and are inexpensive. Some studies using electroactive molecules showed higher detection limits than our results, such as for the detection of human hepatitis B (7.19 nM) [36] and detection of HPV (3.8 nM) [37].

## 4. Conclusions

The importance of oligonucleotide biosensors for hybridization reactions via guanine oxidation has been demonstrated. Oligonucleotide biosensors utilizing pencil graphite for the detection of hybridization are useful for identifying specific nucleotide sequences (dengue virus) with selectivity, providing a convenient and rapid electroanalytical method. The hybridization of oligonucleotides on graphite was used in combination with ADP to obtain information about the hybridization reaction using a guanine oxidation signal. The detection limit for this biosensing electrode with complementary DNA target is found to be 0.92 nM within a hybridization time of 3 minutes.

Therefore, we anticipate that modification of the sequences could be used for the diagnosis of other acute infectious diseases and the utility of new electrochemical hybridization utilizing annealing temperature in the detection of hybridization of oligonucleotides using a biosensor for the diagnosis of diseases is promising. The detection of guanine oxidation eliminates the use of some compounds such as methylene blue, metal complexes and toxic substances as hybridization markers in electrochemical DNA biosensors. Breakthroughs in biosensor techniques and prudent design of diagnostics at the molecular level may contribute to future advancements in type-specific dengue diagnosis.

## Figures and Tables

**Table 1.** Comparison of nucleic acid biosensors under the same immobilization conditions.

**Nucleic acid biosensor**	**Electrode**	**Immobilization Method**	**Electrochemical technique**	**Probe length (mm)**	**Linear range of the hybridization**	**Detection limit**	**Hybridization time**
Hepatitis C virus [38]	Pencil graphite electrode (PGE)	Adsorption	DPV	20	50–750 nM	6.5 nM	15 min
Electrochemical detection of human papilloma virus (HPV) [23]	PGE	Adsorption	SWV	20	—	1.2 ng/μL	3 min
Detection of single nucleotide mutation on p53 [39]	Gold electrode	Adsorption	DPV	15	1–10 nM	0.68 nM	5 min
Oligonucleotide sensors [40]	Carbon past electrode	Adsorption	DPV	20	10–5000 nM	9 nM	5 min
This work	PGE	Adsorption	DPV	18	1–40 nM	0.92 nM	3 min
